# The necrotrophic effector protein SnTox3 re-programs metabolism and elicits a strong defence response in susceptible wheat leaves

**DOI:** 10.1186/s12870-014-0215-5

**Published:** 2014-08-15

**Authors:** Britta Winterberg, Lauren A Du Fall, Xiaomin Song, Dana Pascovici, Natasha Care, Mark Molloy, Stephen Ohms, Peter S Solomon

**Affiliations:** Plant Sciences Division, Research School of Biology, The Australian National University, Canberra, ACT 0200 Australia; Australian Proteome Analysis Facility, Macquarie University, Sydney, NSW 2109 Australia; Molecular Bioscience Division, John Curtin School of Medical Research, The Australian National University, Canberra, ACT 0200 Australia

**Keywords:** Effector triggered susceptibility, *Stagonospora nodorum*, Necrotrophic fungal pathogen, Wheat, Necrotrophic effectors

## Abstract

**Background:**

The fungus *Stagonospora nodorum* is a necrotrophic pathogen of wheat. It causes disease by secreting proteinaceous effectors which interact with proteins encoded by dominant susceptibility genes in the host. The outcome of these interactions results in necrosis, allowing the fungus to thrive on dead plant material. The mechanisms of these effectors though are poorly understood. In this study, we undertake a comprehensive transcriptomics, proteomic and metabolomic approach to understand how a susceptible wheat cultivar responds to exposure to the *Stagonospora nodorum* effector protein SnTox3.

**Results:**

Microarray and proteomic studies revealed that SnTox3 strongly induced responses consistent with those previously associated with classical host defence pathways including the expression of pathogenicity-related proteins and the induction of cell death. Collapse of the photosynthetic machinery was also apparent at the transcriptional and translational level. SnTox3-infiltrated wheat leaves also showed a strong induction of enzymes involved in primary metabolism consistent with increases in hexoses, amino acids and organic acids as determined by primary metabolite profiling. Methionine and homocysteine metabolism was strongly induced upon exposure to SnTox3. Pathogenicity in the presence of homocysteine was inhibited confirming that the compound has a role in plant defence. Consistent with the strong defence responses observed, secondary metabolite profiling revealed the induction of several compounds associated with plant defence, including the phenylpropanoids chlorogenic acid and feruloylquinic acid, and the cyanogenic glucoside dhurrin. Serotonin did not accumulate subsequent to SnTox3 infiltration.

**Conclusions:**

These data support the theory that the SnTox3 effector protein elicits a host cell death response to facilitate the pathogen’s necrotrophic infection cycle. Our data also demonstrate that the mechanism of SnTox3 appears distinct from the previously characterised *Stagonospora nodorum* effector SnToxA. Collectively, this comprehensive analysis has advanced our understanding of necrotrophic effector biology and highlighted the complexity of effector-triggered susceptibility.

**Electronic supplementary material:**

The online version of this article (doi:10.1186/s12870-014-0215-5) contains supplementary material, which is available to authorized users.

## Background

Fungal and bacterial pathogens of plants have been broadly categorised based on their modes of infection and acquisition of nutrients during infection. Classical biotrophic pathogens, such as rusts and mildews, have evolved complex mechanisms and feeding structures to keep their host alive and complete their lifecycles. These pathogens (fungi at least) are typically obligate and host specific. To cause disease, these biotrophic pathogens must avoid the host’s innate immune system which detects common pathogen signals such as chitin or flaggelin [1,2]. This is achieved through the secretion of effector molecules which interfere with the innate immune system resulting in the pathogen remaining “hidden” from the plant. Plants though have evolved a second line of defence known as Effector Triggered Immunity (ETI) whereby these specific effector molecules are recognised by host resistance (R) genes leading to localised cell death and suppression of biotrophic growth.

Necrotrophic pathogens in contrast rapidly kill their host to infect and reproduce. Historically it was assumed that the pathogen secreted lytic and cell wall degrading enzymes to degrade host tissue and release nutrients. Recent studies though have revealed that at least some necrotrophic pathogens hijack the host’s own cell death machinery rather than rely solely on lytic enzymes. These pathogens actively secrete effector molecules that are detected by R-like genes to trigger hypersensitive response pathways and initiate cell death. This phenomenon is known as dominant susceptibility [3].

The best-described example of dominant susceptibility is that of *Cochliobolus victoriae*, the causal agent of victoria blight of oats. Pathogenic strains synthesize the chlorinated cyclic pentapeptide victorin [4]. Rapid progress in dissecting the mechanism of victorin-induced cell death was possible as *C. victoriae* is also able to infect *Arabidopsis thaliana*. Using the genetic tools available for this model plant, it was demonstrated that the activity of victorin was dependent on LOV1, a protein closely resembling the nucleotide-binding site-leucine-rich repeat (NBS-LRR) resistance proteins [5]. It was more recently shown that the interaction of victorin with LOV1 occurred through its binding to the thioredoxin TRX-h5 leading to a resistance like response and disease susceptibility to *C. victoriae* [6]. Based on this, it was proposed that victorin mimics a conventional (biotrophic) effector that elicits a cell death response which can be exploited by the necrotrophic *C. victoriae*. This was the first demonstrated example of a pathogen effector protein interacting with a host R-like protein giving rise to gene-for-gene dominant susceptibility (in contrast to the typically reported dominant resistance).

Other cases of host-specific necrotrophic effectors are beginning to emerge. For example, *Pyrenophora tritici-repentis*, the causal agent of wheat tan spot, produces the proteinaceous effector ToxA [7]. ToxA is a small (13.2 kDA) secreted protein [8–10] that leads to necrosis in ToxA-sensitive wheat lines. ToxA is internalised into plant mesophyll cells subsequent to the pathogen penetrating the leaf [11] and interacts with the chloroplast-localized protein ToxA-BP1 leading to collapse of photosynthesis, the accumulation of reactive oxygen species (ROS) and eventually to cell death [12,13]. As demonstrated with victorin, ToxA is indirectly recognised by an NBS-LRR protein named Tsn1 [14]. Transcriptomic [15], proteomic and metabolomic [16,17] analyses of ToxA-triggered responses in wheat have shown that ToxA invokes a variety of host responses including the induction of defence.

The wheat pathogen *Stagonospora nodorum* is another necrotroph shown to infect through a gene-for-gene mechanism [18–21]. *S. nodorum* has emerged as one of the model organisms for studying plant – necrotrophic pathogen interactions due to its genetic tractability and extensive functional genomics resources [21,22]. Three proteinaceous effectors, SnToxA [22], SnTox1 [23] and SnTox3 [3] have recently been identified and are required for disease. *SnToxA* was first identified through the sequencing of the *S. nodorum* genome. The nucleotide sequence was found to be almost identical to *ToxA* from *P. tritici-repentis* and subsequent analysis has provided strong evidence that *SnToxA* was laterally transferred from *S. nodorum* to *P. tritici-repentis* shortly prior to 1940 [22]. Recent mode-of-action analyses have proven that SnToxA and ToxA share identical functions [16].

The second effector gene identified in *S. nodorum* was *SnTox3* [3]. The protein is encoded by a 693-bp intron-free gene that shares no homology to other proteins. Expression of *SnTox3* in *S. nodorum* is induced during the early infection stages [24]. It is synthesized as a prepropeptide and cleavage of the signal peptide and the prosequence leads to maturation of the small (18 kDa) effector [3,19]. Three disulfide bridges formed by six cysteine residues in SnTox3 are essential for structure and function of the effector protein. QTL analysis has shown that two homoeologous wheat susceptibility genes, *Snn3-B1* and *Snn3-D1*, are involved in SnTox3 sensitivity [25].

As part of a larger study in our laboratory to understand the roles and mechanisms of necrotrophic effectors, we are using functional genomics tools to identify the pathways and mechanisms these effectors exploit to cause disease. Unlike *C. vitoriae*, *S. nodorum* is strictly host specific for wheat and does not infect *A. thaliana*, thus the genetic resources for dissecting the mechanisms of SnToxA and SnTox3 are limited. In this study, gene expression, protein abundance and metabolite levels have all been examined in susceptible wheat infiltrated with SnTox3. This approach has identified a number of key host pathways that are involved in symptom development upon exposure to SnTox3 and provided further insight into the complex *S. nodorum-*wheat interaction.

## Methods

### Plant and fungal material

SnTox3-sensitive wheat (*Triticum aestivum* genotype BG220) and the *S. nodorum* wildtype strain SN15 were grown as previously described [26].

### Detached leaf assay

Wheat leaf segments were placed on water agar plates containing 0.03% benzamidazole and 300 μM homocysteine (6 leaves/plate). 5 μL of a *S. nodorum* spore suspension with 10^6^ spores mL^−1^ in 0.02% Tween 20 were spotted onto the leaf surface and kept at 20°C for seven days. Leaves were ranked by increasing symptom severity and analysed using a Kruskal-Wallis test to determine statistical significance. The experiment was performed in five replicates.

### Isolation of SnTox3 from *Pichia pastoris*

The *Pichia pastoris* strain ×33 was transformed with the empty vector pGAP2A and pGAP2A containing the coding sequence of SnTox3. Strains expressing His-tagged SnTox3 and control strains containing an empty vector were inoculated into YPD medium (10 g L^−1^ yeast extract, 20 g L^−1^ peptone, 20 g L^−1^ glucose) and incubated for 4 days at 30°C with shaking (300 rpm). Culture supernatants were collected by centrifugation (10 min, 4000 rpm, 4°C) and frozen in liquid nitrogen before lyophilisation. The lyophilized material was resuspended in a minimal amount of sterile dH_2_O and dialyzed (6–8000 MWCO, Spectrum Laboratories, Inc.) against water overnight. For transcriptome and proteome analysis, the dialyzed supernatants were further purified using PD-10 desalting columns (GE Healthcare) and water as eluate.

For metabolome experiments, the dialysed and resuspended culture supernatants were diluted 1:2 in freshly prepared 2× binding buffer (20 mM NaPO_4_, 500 mM NaCl, 20 mM imidazole pH 7.4). SnTox3 and the empty vector control were then purified on an AKTA Explorer FPLC system and a 5 mL HisTrap FF affinity column (GE Healthcare, Amersham Biosciences). Samples were loaded at 5 mL min^−1^ followed by a column wash with binding buffer for 25 column volumes at 1 mL min^−1^ and eluted with 5 column volumes of 100% elution buffer (20 mM NaPO_4_, 500 mM NaCl, 500 mM imidazole, pH 7.4). Fractions eluting at 280 nm were pooled and dialyzed against water.

### Viability staining using Trypan blue

Leave segments were incubated at 99°C for 5 min in staining solution containing 2 mL Trypan blue (0.4%) (Invitrogen), 5 mL glycerol (70% w/v), 5 mL phenol and 3 mL of water. Leaves were destained over night at room temperature in chloral hydrate (2.5 g/mL) and stored in 70% glycerol. Stained samples were examined using an Axioplan universal microscope (Zeiss, Germany) and 10× maginification.

### Microarray analysis

13 day-old seedlings were syringe-infiltrated with dialyzed SnTox3 or empty vector-control. Samples for microarray analysis were collected in biological triplicates at 6, 12, 24 and 48 hpi and frozen in liquid nitrogen. Total RNA was extracted using the Spectrum Plant Total RNA kit (Sigma) and the quality analysed using the Agilent Bioanalyzer 2100 with an Agilent RNA 600 Nano Kit. Labelling and hybridisation to Affymetrix GeneChip Wheat Genome arrays was performed at the Ramaciotti Centre for Gene Function Analysis. The resulting data files are available through the Gene Expression Omnibus at NCBI (Accession number GSE59723).

Following microarray hybridizations, CEL files were imported into Partek Genomics Suite Version 6.3 (Partek, St Louis, Missouri, USA) using the following parameters: Pre-background Adjustment: Adjust for GC Content; Background Correction: RMA Background Correction; Quantile Normalization: Quantile Normalization; Log Probes using Base: 2; Probeset Summarization: Mean. Microarray quality was assessed by a PCA plot and the quality-control metrics implemented in Partek Genomics Suite. Based on these, the quality of all array hybridizations was assessed as acceptable. The experiment was analysed in Partek GS using a 2-way ANOVA model based on the method of moments [27]. Model: Y_ijk_ = μ + Treatment_i_ + Timepoint_j_ + Treatment * Timepoint_ij_ + ε_ijk_. Y_ijk_ represents the kth observation on the ith treatment at the jth timepoint μ is the common effect for the whole experiment. ε_ijk_ represents the random error present in the kth observation on the ith treatment at the jth timepoint. The errors ε_ijk_ were assumed to be normally and independently distributed with mean 0 and standard deviation δ for all measurements.

SnTox3- and empty vector-treated samples at each individual time point were compared as contrasts [28]. A FDR threshold of 0.001 was applied for the identification of significantly differentially expressed probe sets. Probe sets with a fold change greater or less than 2 in the comparison of SnTox3- vs. empty vector-infiltrated plants were considered as transcriptionally induced or repressed respectively.

Annotation of differentially regulated probe sets was based on [15]. MapMan [29] was used to assign regulated probe sets to molecular functions.

### Protein extraction

Six leaves from each sample (about 150 to 200 mg) were chopped into 2–3 mm pieces and placed into Precellys tubes CK28R with 1 mL of ice cold TCA-acetone precipitation buffer [10% TCA in acetone] and homogenised using Precellys for 45 seconds at 6500 rpm. The homogenised samples were placed into −20°C freezer for 1.5 hr and then centrifuged at 4500 g, 5°C for 45 min. The supernatant was discarded and the pellet mixed with 1 mL of ice cold acetone, left overnight at −20°C and then centrifuged at 4000 g, 5°C for 30 min and the supernatant removed. This acetone precipitation procedure was repeated three times.

To solubilize proteins, 400 μL of lysis buffer [50 mM HEPES, 2% SDS, pH 7.5, with protease inhibitor] was added to pellets, homogenised using Precellys at 6000 rpm for 20 sec, cooled on ice for 5 min, sonicated for 10 minutes, centrifuged at 12000 g for 10 minutes. This protein extraction procedure was repeated once. Supernatants from the two extractions were pooled.

The protein extraction supernatant was buffer exchanged using a 5KDa molecular weight cut-off spin filter (VS0212, VIVA Science) into iTRAQ sample buffer [0.25 M TEAB (triethylammonium bicarbonate), 0.05% SDS, pH 8.5]. A protein assay was carried out using Direct Detect (Millipore).

### iTRAQ experimental design and procedures

The experiment comprised 24 samples from a time course experiment on empty vector (EV) and SnTox3 infiltrated samples at 12, 24, 48 and 72 hpi. The 24 samples and a common reference pooled sample (made by taking an equal amount of protein from the EV 12 and EV 24 samples) were placed on eight 4-plex iTRAQ runs as described in Additional file 1. The iTRAQ experimental procedures were described in our previous paper [30]. Briefly, for each iTRAQ run, four protein samples were digested with trypsin followed by labelling with iTRAQ 4-plex tags. The four labelled samples were then mixed and fractionated into 12 fractions by strong cation exchange (SCX) HPLC. Each SCX fraction was then subjected to reverse phase nanoLC ESI MS/MS data acquisition.

### Database search and protein identification

The experimental nanoLC ESI MS/MS data were submitted to ProteinPilot V4.2 (AB Sciex) for data processing using a combined cereals database assembled by combining available wheat identifiers from Uniprot and NCBI, *Brachypodium* identifiers (downloaded from http://www.brachypodium.org/), Sorghum, Maize and Rice identifiers as well as Uniprot and TrEMBL sources, for a total of 299,040 proteins [30]. The search parameters took into consideration cysteine modification by methyl methanethiosulfonate, digestion of peptides with trypsin and default biological modification settings. False Discovery Rate (FDR) Analysis was enabled. Proteins identified with better than 1.3 *Unused* score (greater than 95% protein identification confidence) were used for further data analysis. In addition to the protein detection confidence cutoff an overall false discovery rate was estimated based on the percentage of decoy database hits (reversed hits).

### Proteomic data analysis

The data from all eight runs was assembled using the pooled sample as a common reference for ratio calculation. A series of pair-wise comparisons of biological interest were undertaken separately for each protein, across the time course: TOX and empty vector protein ratios at 12 hours, then respectively 24, 48 and 72 hours. The ratios were compared in each case by a two sample *t*-test of log abundance ratios to the pool; proteins identified in all triplicate samples for the respective comparison and having a *t*-test p-value < 0.05 and a fold change > 1.2 or < 0.83 were regarded as being differentially expressed across the time course. The fold change cut-offs selected were necessarily lower than for the transcriptomics approach due to iTRAQ ratio compression, hence their impact on false discovery rates across biological replicates was assessed and found to be low for all runs [Additional file 2]. Overall fold changes between TOX and empty vector samples at each particular time point were calculated as ratios of geometric means across the replicates. All proteomics mass spectrometry data can be found at ftp://ftp.proteome.org.au/WheatANU/.

### Metabolite profiling

All GC-MS and LC-MS metabolite profiling of whole leaf material and data analysis performed in this study were as per previously described [16,17]. For GC-MS of apoplastic fluid, apoplast was extracted as described previously [31]. Cytosolic contamination of the apoplast was assessed by measuring for glucose 6-phosphate dehydrogenase activity [31]. Cold methanol (225 μL, 100%) was added to the apoplastic fluid (25 μL) to precipitate proteins overnight at −20°C. Methanol extracts were centrifuged (5 min, 10,000 g) and supernatant dried down to completeness.

## Results

### Microarray profiling reveals strong gene expression changes upon SnTox3 infiltration

**Figure 1:**
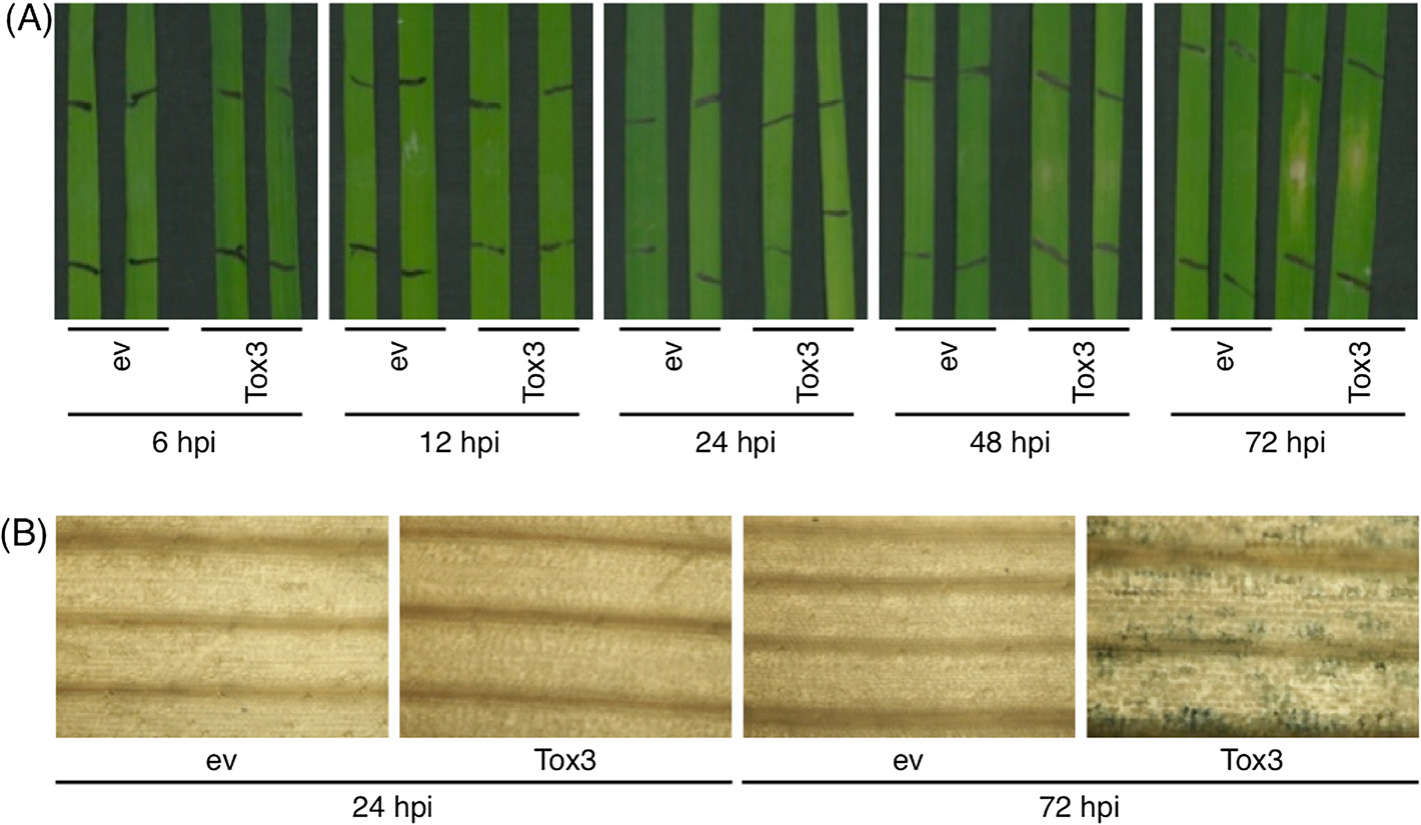
**Tox3-infiltration induces necrosis and cell death in the wheat line BG220. (A)** 13-day old BG220 seedlings were infiltrated with SnTox3 or the empty vector control (ev). Necrotic lesions became visible 48 hpi in SnTox3-infiltrated plants. **(B)** Trypan blue staining of empty vector- and SnTox3-infiltrated leave areas for visualization of cell death.

For the identification of differentially expressed genes, SnTox3 was expressed in *Pichia pastoris* and purified from culture supernatants. Supernatants of empty vector-transformed yeast cultures served as control. 13 day-old seedlings of the wheat variety BG220 harbouring the susceptibility gene Snn3 were infiltrated with purified SnTox3 and the empty vector control (ev) (Figure 1A). Symptoms of necrosis became visible 48 hours post infiltration (hpi) in SnTox3-infiltrated leaves. Viability staining with Trypan blue showed cell death 72 h post infiltration with SnTox3 (Figure 1B).

**Figure 2:**
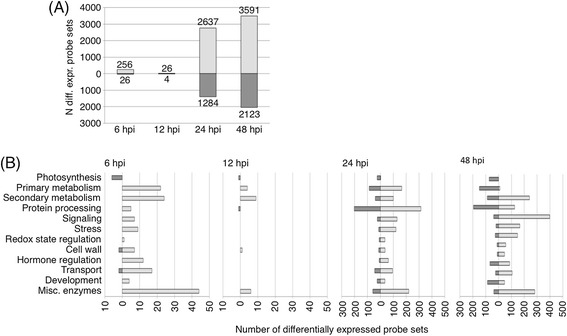
**SnTox3 infiltration leads to strong significant changes in the wheat transcriptome. (A)** Number of statistically significant differentially regulated microarray probe sets that are induced (light grey) or repressed (dark grey) in response to SnTox3 infiltration 6, 12, 24 and 48 hours post infiltration (hpi). **(B)** MapMan analysis of differentially regulated probe sets was performed to group induced (light grey) or repressed (dark grey) probe sets into functional categories. As less probe sets were differentially regulated at 6 and 12 hpi, a different scale was chosen for the graphical representation of the later time points.

Affymetrix microarray analysis was undertaken at six, 12, 24 and 48 hpi to analyse the gene expression in wheat upon SnTox3 exposure. More than 6400 probe sets, coding for 5700 genes, showed differential expression (fold change >2, *p* < 0.05) at at least one of the time points when comparing SnTox3- to mock infiltrated plants (Figure 2A; Additional files 3 and 4).

The differentially regulated probe sets were analysed using MapMan to classify gene functions (Figure 2B). The majority of probe sets could not be assigned to a functional category. Ninety-eight probe sets associated with photosynthesis showed down-regulation at all time points consistent with the visible collapse of plant tissue and chlorosis upon SnTox3 infiltration. Twelve probe sets were up-regulated at the same timpoints. Genes involved in primary and secondary metabolism showed induction at all time points. Probe sets grouped into the MapMan bins ‘Signalling’, ‘Stress’, ‘Redox state regulation’ and ‘Cell wall’ were also up-regulated, indicating the induction of plant defence responses.

Many genes typically associated with plant defence responses showed strong induction 24 and 48 hpi (Table 1). Of the 25 probe sets with the highest fold change induced at both 24 and 48 hpi, 19 were involved in plant defence mechanisms including PR proteins and components of the jasmonic acid (JA) pathway and phenylpropanoid pathways. Induction of phenylpropanoid biosynthesis seems to be among the initial defence responses. Of the 30 probe sets differentially regulated at 12 hpi, eight encode for phenylalanine ammonia lyases which catalyse the first committed enzymatic reaction of phenylpropanoid biosynthesis. Expression of these probe sets remained highly induced (up to 70-fold induction) throughout the time course.

**Table 1:** **SnTox3 induces expression of plant defence responses**

**Probe set ID**	**Fold-change 24 hpi**	**Fold- change 48 hpi**	**Annotation**	
Ta.278.1.s1_x_at	35.124	168.094	expressed protein	PR-1
Ta.278.1.s1_at	28.26	151.169	expressed protein	
TaAffx.15327.1.s1_at	45.891	193.677	glucan endo-1,3-beta-glucosidase GII precursor, putative, expressed	PR-2
Ta.20121.1.s1_x_at	19.018	76.22	glucan endo-1,3-beta-glucosidase, acidic isoform precursor, putative	
Ta.28.1.s1_at	28.122	129.871	glucan endo-1,3-beta-glucosidase, acidic isoform precursor, putative	
Ta.21342.1.s1_x_at	30.836	158.506	basic endochitinase 1 precursor, putative, expressed	PR-3
Ta.2784.1.a1_at	12.926	100.179	acidic endochitinase Q precursor, putative, expressed	
Ta.14946.1.s1_at	23.018	79.238	acidic endochitinase Q precursor, putative, expressed	
Ta.9226.1.s1_at	33.843	199.463	win2 precursor, putative, expressed	PR-4
Ta.24501.1.s1_at	21.754	75.609	thaumatin-like protein precursor, putative, expressed	PR-5
Ta.23322.1.s1_s_at	15.541	72.758	protein P21, putative, expressed	
Ta.13371.1.s1_at	23.75	81.92	Bowman-Birk type bran trypsin inhibitor precursor, putative, expressed	PR-6
Ta.169.1.s1_x_at	33.72	78.158	germin-like protein 2b	PR-15
Ta.8447.1.s1_a_at	28.453	131.591	cytochrome P450 76C2, putative, expressed	cyto-chrome P450
Ta.8447.1.s1_x_at	23.447	66.609	cytochrome P450 76C2, putative, expressed	
Ta.22615.1.s1_at	23.678	130.487	cytochrome P450 71D6, putative, expressed	
TaAffx.109794.1.s1_at	32.04	72.503	cytochrome P450 76C1, putative	
Ta.21307.1.s1_x_at	89.44	116.551	peroxidase 12 precursor, putative, expressed	phenyl-propanoid pathway
Ta.22564.2.s1_a_at	59.78	79.389	peroxidase precursor, putative, expressed	
Ta.82.1.s1_at	57.636	77.723	peroxidase precursor, putative, expressed	
Ta.7022.1.s1_s_at	66.367	72.208	phenylalanine ammonia-lyase, putative, expressed	
Ta.30827.1.a1_x_at	20.382	78.043	jasmonate-induced protein, putative, expressed	jasmonic acid
Ta.23763.1.s1_at	39.084	80.337	lipoxygenase 1, putative, expressed	
Ta.23327.1.s1_at	37.873	101.178	NA	
TaAffx.26815.1.s1_at	74.671	88.734	NA	

SnTox3 also induced significant gene expression changes in various signal transduction pathways. 147 probe sets annotated as kinases and 72 as transcription factors were differentially regulated at both 24 and 48 hpi (Additional file 5). Of the 93 probe sets on the microarray chip encoding for receptor-like kinases (RLK), 22 (24%) were up-regulated at 24 and 48 hpi. Among the differentially regulated kinases, 13 probe sets (coding for 10 genes) were highly induced with a fold change greater than 10. Three probe sets representing transcription factors also exhibited a greater than 10-fold induction at these later time points.

### Proteome profiling of SnTox3-treated leaves reveals significant changes in primary metabolism and photosynthetic proteins

**Figure 3:**
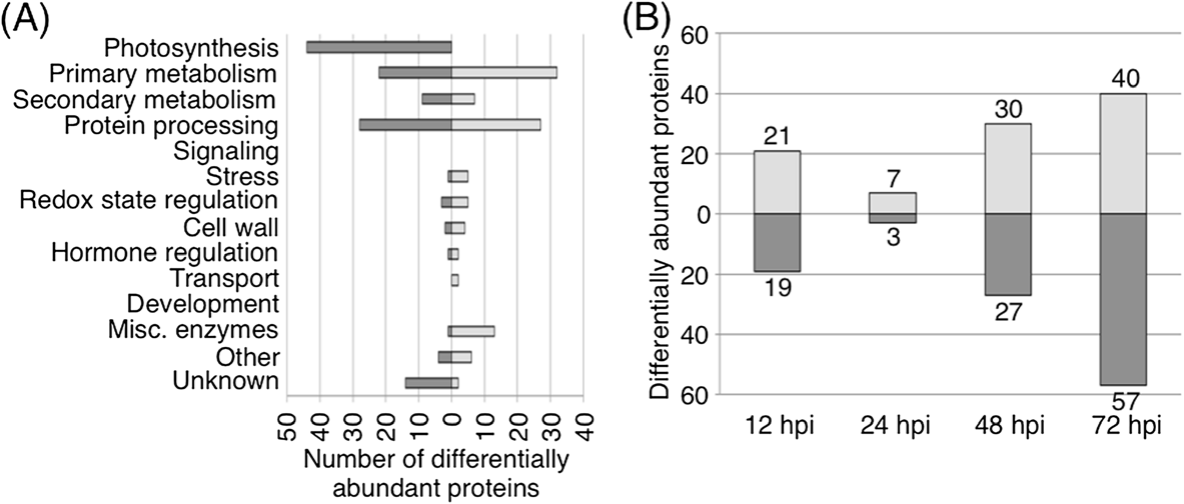
**SnTox3-induces changes in protein abundance. (A)** Number of differentially expressed proteins in functional groups that show higher (light grey) or lower (dark grey) protein abundance in SnTox3-treated plants compared to mock–infiltrated BG220 seedlings. **(B)** Number of proteins with induced (light grey) or repressed (dark grey) protein levels in SnTox3-infiltrated plants over time.

iTRAQ-based proteomics was used to identify changes to the wheat leaf proteome subsequent to SnTox3 exposure. 172 wheat proteins were identified as being differentially abundant at at least one time point in SnTox3-infiltrated plants compared to empty vector controls (*p* <0.05, average fold change < 0.83 and > 1.2 for down- and up-regulated proteins, respectively; Additional file 6). 143 of these proteins have a known annotation, and for 135, a function could be predicted (Figure 3A). BLAST searches were performed to identify corresponding microarray probe sets coding for the regulated proteins (www.plexdb.org). For 105 proteins (61%), no change in transcript abundance was identified subsequent to SnTox3 exposure justifying the proteomics approach to identify differentially regulated processes.

Many (35%) of the repressed proteins were predicted to be involved in photosynthesis (Figure 3A). Several enzymes involved in primary metabolism were more abundant in SnTox3-treated plants, including enzymes of the pentose phosphate pathway. 6-phosphogluconate dehydrogenase was induced 1.4 and 1.5-fold at 24 and 72 hpi, respectively whilst transaldolase was 1.6-fold higher at 72 hpi. Two glycolytic enzymes, phosphofructokinase (×1.4 at 48 hpi) and 2,3-bisphosphoglycerate-independent phosphoglycerate mutase (×1.5 at 72 hpi) were also present at higher levels after SnTox3 infiltration, whilst fructose 1,6-bisphosphate aldolase (EC: 4.1.2.13) showed slightly reduced protein levels (×0.82 at 72 hpi).

There were other notable changes identified through this proteomics approach. 3-Ketoacyl-CoA thiolase, catalysing the last enzymatic step in β-oxidation, was significantly more abundant at 48 and 72 hpi (×2.0 and 1.3, respectively) indicating increased lipid catabolism. Phospho-2-dehydro-3-deoxyheptonate aldolase, a key enzyme of the shikimate pathway, was also significantly more abundant (×1.5) subsequent to SnTox3 infiltration (48 hpi) suggesting an induction in aromatic amino acid biosynthesis. Other proteins involved in amino acid biosynthesis were less abundant. Expression of the enzymes diaminopimelate decarboxylase (lysine), 2-isopropylmalate synthase (leucine) and ketol-acid reductoisomerase (valine, leucine and isoleucine) were repressed during the latter stages of SnTox3 treatment. It was also observed that the abundance of the PtrToxA binding protein ToxABP1 (Q5XUV3_WHEAT) was significantly decreased after SnTox3 treatment with a fold change of 0.713.

The process of iTRAQ data acquisition leads to an underestimation of true iTRAQ ratios, (for example true fold changes of 1.5, 2 and 3 were found to correspond to detected fold changes of approximately 1.3, 1.5 and 2 [32]) thus we can conclude that the biological fold-changes are likely larger than the observed iTRAQ ratios reported here (please refer to Additional file 2 for further details).

### SnTox3 induces methionine metabolism

**Figure 4:**
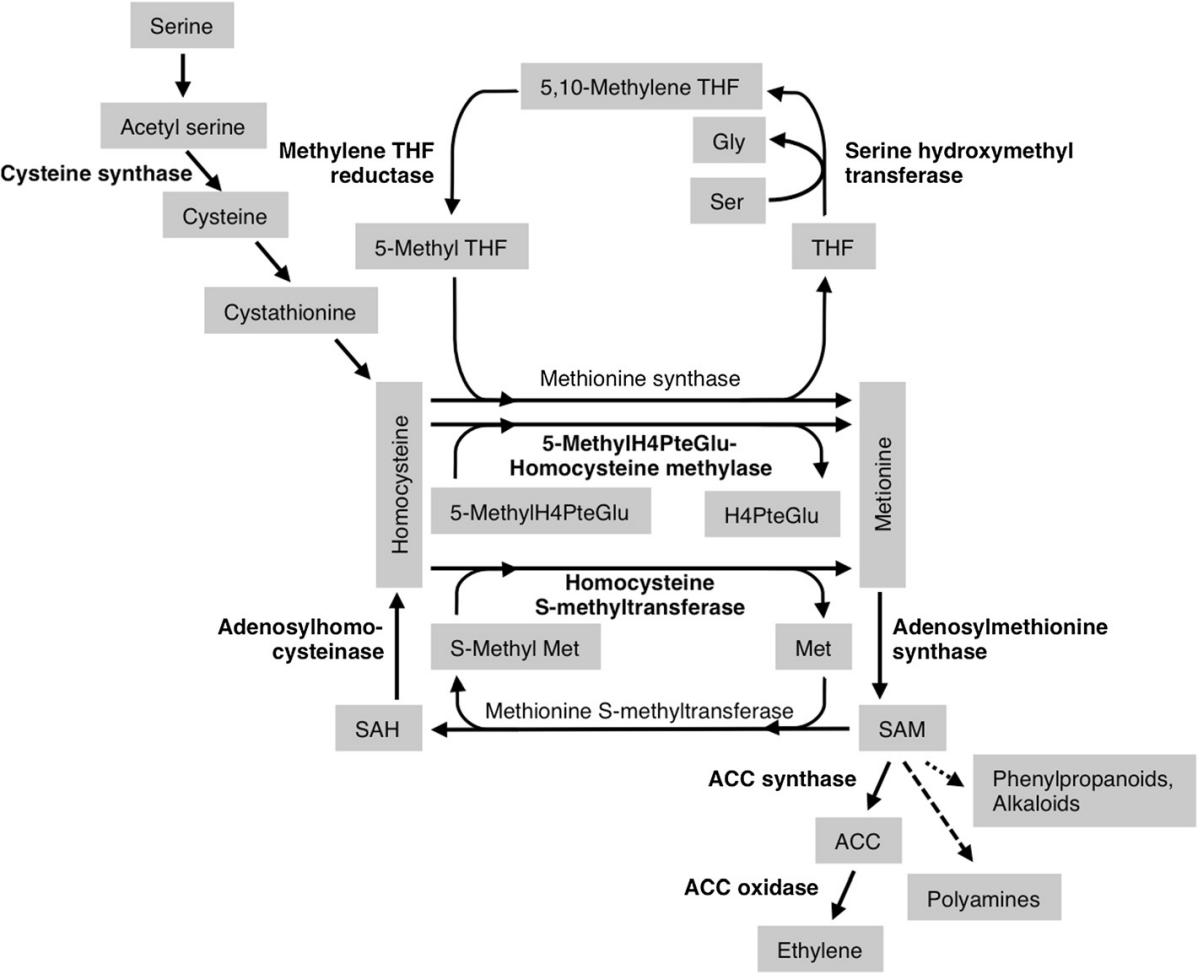
**SnTox3 induces expression of enzymes involved in methionine metabolism.** Enzymes written in bold are induced by SnTox3 THF: tetrahydrofolate, Gly: glycine, Ser: serine, H4PteGlu: tetrahydropteroyltriglutamate, Met: methionine, SAM: S-adenosyl methionine, SAH: S-adenosyl homocysteine, ACC: aminocyclopropane carboxylate SAM is a methyl donor for phenylpropanoid biosynthesis (⤑) and precursor of polyamines (⤍).

Careful scrutiny of both the microarray and proteomics data revealed a striking impact on the methionine biosynthetic pathway upon infiltration with SnTox3 (Figure 4, Table 2). Expression of a probe set encoding a cysteine synthase was 3.7 fold induced upon SnTox3-infiltration. Enzymes catalysing the recycling of 5-methyl tetrahydrofolate were also up-regulated at the transcriptional and translational level. 4 probe sets encoding for serine hydroxymethyl transferases were induced (×2.5-2.9) as was the expression of methylene THF reductase genes (×2.2 and 4.1) leading to higher abundance of the protein (×1.6). The second enzyme involved in methionine production is 5-methyl tetrahydropteroyltriglutamate-homocysteine methylase. Eight probe sets coding for this enzyme showed strong up-regulation in response to SnTox3 (×3.4-14.1), whilst the abundance of two of the resulting proteins was also increased (×2.0 and 2.6). Thirdly, homocysteine S-methyltransferase, which catalyses synthesis of two methionine molecules from homocysteine and S-methyl methionine, was highly induced at the transcriptional level (×2.6-20.1 of 5 probe sets).

**Table 2:** **SnTox3 induced up-regulation of genes and proteins involved in methionine metabolism**

		**Transcriptome (48 hpi)**		**Proteome (72 hpi)**	
	**EC number**	**Probe sets**	**Fold change**	**Proteins**	**Fold change**
Cystathionine b-lyase	4.4.1.8	1	3.7		
Serine hydroxymethyl transferase	2.1.2.1	4	2.5-2.9	1	0.82
Methylene THF reductase	1.5.1.20	2	2.2-4.1	1	1.6
5-methylH4PteGlu-homocysteine methylase	2.1.1.14	8	3.4-14.1	2	2.0-2.6
Homocysteine S-methyltransferase	2.1.1.10	5	2.6-20.1		
Adenosylmethionine synthase	2.5.1.6	3	2.5-3.2	1	2
Adenosylhomocysteinase	3.3.1.1.	1	2.6	1	2.1
ACC synthase	4.4.1.14	2	5.2-11.8		
ACC oxidase	1.14.17.4	2	3.6		

Numbers of differentially regulated probe sets and proteins are given with their fold change range. THF: tetrahydrofolate, H4PteGlu: tetrahydropteroyltrigultamate.

Adenosylmethionine synthase, which catalyses the reaction from methionine to S-adenosyl methionine (SAM), responded positively at the transcriptional (×2.5-3.2) and translational level (×2.0) upon SnTox3 exposure. The expression of adenosylhomocysteinase was also induced by SnTox3 (×2.6 fold). Genes encoding aminocyclopropane (ACC) synthase and ACC oxidase, both involved in the synthesis of ethylene from SAM, were up-regulated in SnTox3-infiltrated plants (×2.0-11.8).

**Figure 5:**
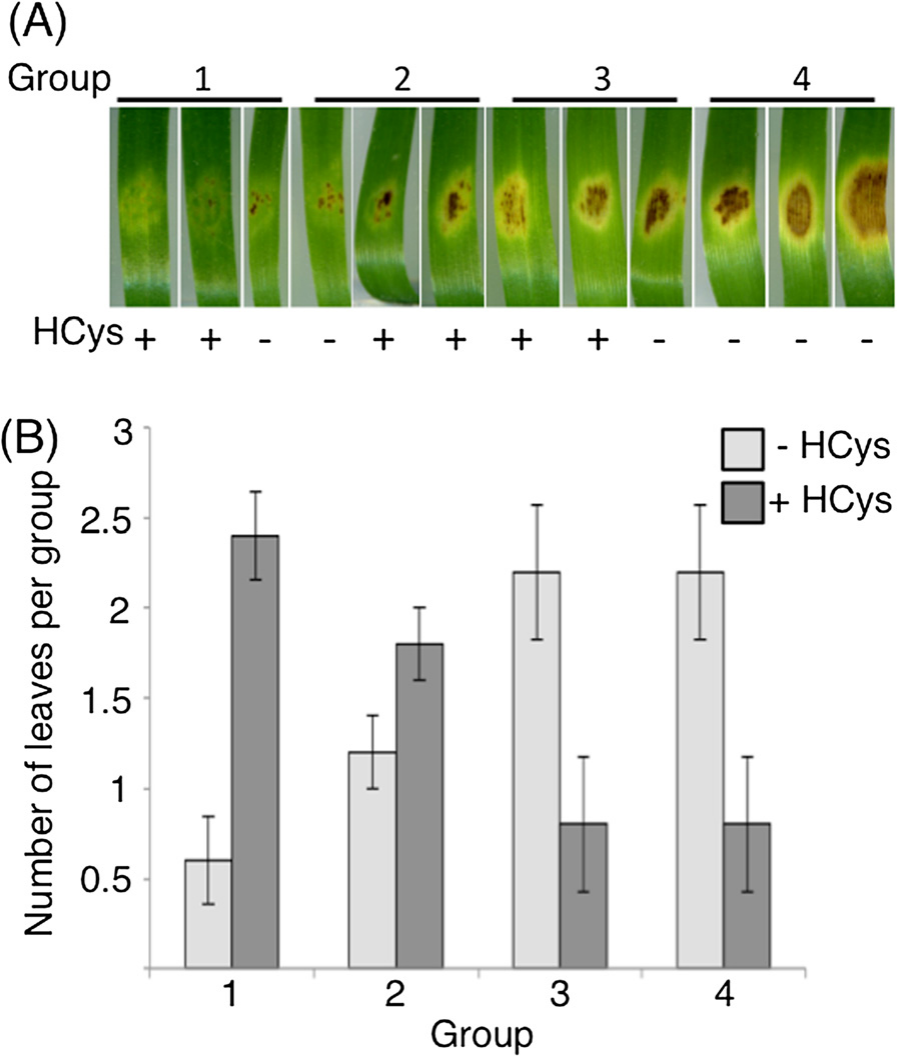
**Exposure of** ***S. nodorum*** **to homocysteine during infection leads to reduced virulence. (A)** Detached leaf assays were performed on agar plates in the absence (−) or presence (+) of 300 μM homocysteine. Six leaves for each treatment were inoculated with 5000 spores and ranked according to the severity of symptom development 7 days post inoculation. Based on increasing symptom severity, the infected leaves were assigned to 4 groups of three leaves each. Average numbers of leaves in each of the groups are given in **(B)**. Error bars indicate the standard error of five replicates.

Given this striking up-regulation of the homocysteine metabolism, we analysed whether or not it played a role in disease development. Detached wheat leaves were inoculated with *S. nodorum* in the presence or absence of 300 μM homocysteine and scored for disease seven days later (Figure 5). In the absence of homocysteine, large necrotic lesions were evident one week post inoculation. In the presence of homocysteine, the severity of the lesions was significantly less with many of the replicates failing to develop large necrotic lesions. In many instances, only mild chlorosis or necrotic flecking was evident suggesting that the presence of homocysteine was inhibitory to disease development. The severity of the symptoms in leaves treated with homocysteine was significantly lower than untreated controls as determined by a Kruskal-Wallis test (p-value = 0.01046). To determine if this effect on growth by homocysteine was *in planta* specific, the growth of *S. nodorum* was measured in vitro in varying concentrations of homocysteine. No effect on growth was observed suggesting that the reduction in pathogenicity was not directly due to the increased homocysteine levels (Additional file 7), but more likely to enhanced downstream responses such as ethylene signalling and/or defence compound synthesis.

### Primary metabolite profiling demonstrates an accumulation of sugars and organic acids in SnTox3-treated wheat

**Figure 6:**
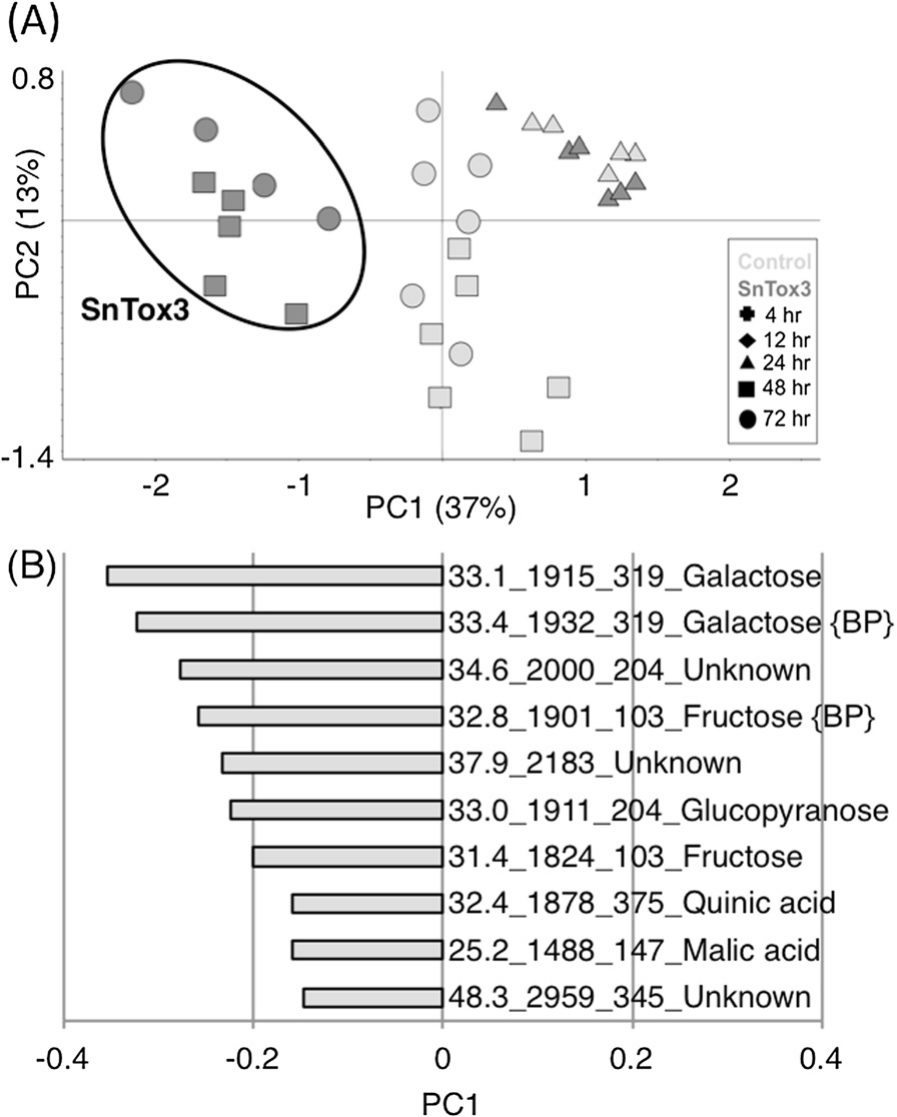
**Metabolite profiling of whole leaf extract from SnTox3 infiltrated wheat.** GC-MS analysis of polar metabolites in wheat leaves infiltrated with SnTox3 or controls at various time points post infiltration. **(A)** Principal component analysis (PCA) of data showing variation amongst the metabolite content of samples and replicates. The samples containing SnTox3-infiltrated wheat extracts at 48 and 72 hpi are circled to highlight the clear differentiation in metabolite content compared to controls at these time points. This difference is best described by principal component 1 (PC1). **(B)** Metabolite loadings of PC1 illustrating ten metabolites with the highest contribution to the differences between control and SnTox3 infiltrated wheat at 48 and 72 hpi. All ten metabolites were present at a higher abundance in SnTox3-infiltrated wheat at these time points.

Gas chromatography–mass spectrometry (GC-MS) was used to identify metabolites whose abundance is influenced by SnTox3. Principal component analysis (PCA) of the GC-MS data demonstrated clustering of the biological replicates verifying the experimental design and robustness of the method. The largest variation was represented by principal component 1 (PC1) which accounted for 37% of the variance in the data set distinguishing the 48 and 72 hpi SnTox3-infiltrated samples from controls and earlier time points (Figure 6A). The metabolite loadings for the 10 mass features with the highest contribution to PC1 are detailed in Figure 6B. The majority of metabolites contributing to the variance in the data set were more abundant in the SnTox3-infiltrated wheat.

In order to determine significant metabolite changes in wheat induced by SnTox3, we performed univariate and multivariate statistical analyses on the GC-MS dataset (Additional file 8). Thirty-nine mass features were identified to be significantly altered in SnTox3-treated wheat relative to the control; 30 of these increased in abundance. Putative metabolite identifications were established by searching the Golm Metabolome Database and the NIST08 library with 16 confirmed with an in-house database. The remaining metabolites were unidentified.

A number of sugars showed an increased abundance in SnTox3-infiltrated plants including hexoses, pentoses and di- and tri-saccharides. Fructose, glucose, galactose and ribose demonstrated the largest fold changes (4–5) at 48 and 72 hpi. Glyceric acid, malic acid, 2-keto-gluconic acid and erythronic acid were more abundant in SnTox3 infiltrated plants, while N-acetylglutamic acid was less abundant. An unidentified compound with a retention time of 31.4 minutes showed the largest change of 11 fold higher in SnTox3 infiltrated plants at 72 hpi.

A comparable approach was also undertaken to analyse the metabolite content of the apoplast in SnTox3-infiltrated wheat (Additional file 9; Additional file 10). The majority of the metabolites that were more abundant subsequent to SnTox3 infiltration were organic acids, sugars and amino acids. All of the identified sugars including sucrose, glucose, fructose and raffinose were all more abundant in SnTox3-infiltrated wheat over the time course as were all three identified amino acids, glutamine, threonine and serine. The largest abundance changes at four hpi were observed for glutamic acid and glucopyranose, which increased 3–4 fold in response to SnTox3. The largest significant decrease in a metabolite at four hpi was pyruvic acid, which decreased 2 fold. At 72 hours the largest increases of 5–6 fold were seen in glutamic acid, citric acid and quinic acid. At 72 hours, the largest decreases in metabolites in SnTox3 wheat were all unknown metabolites. Cytosolic contamination of the apoplast samples was negligible as determined by assaying for glucose 6-phosphate dehydrogenase activity (data not shown).

### SnTox3 infiltration induces extensive changes in wheat secondary metabolism

**Figure 7:**
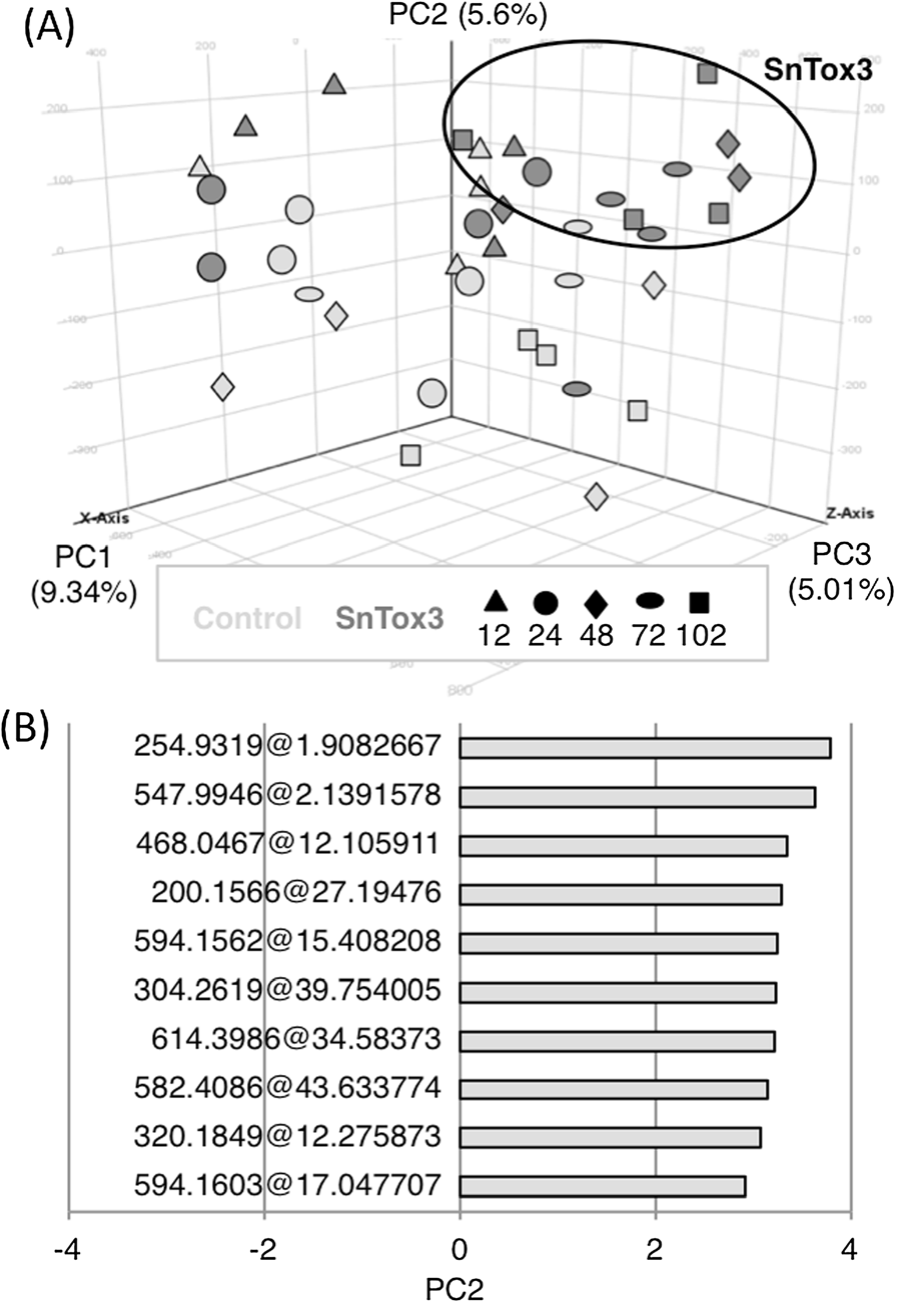
**Metabolite profiling of whole leaf extract from SnTox3 infiltrated wheat.** LC-MS analysis of semi-polar metabolites in leaves infiltrated with SnTox3 or controls at various time points post infiltration. (A) Principal component analysis (PCA) of data showing variation amongst samples and replicates. The SnTox3-infiltrated wheat samples at 48–102 hpi are circled to highlight the separation relative to controls, which indicates a difference in metabolite content at these latter time points. This difference is best described by principal component 2 (PC2). **(B)** Metabolite loadings of PC2 illustrating the ten metabolites with the highest contribution to the differences between control and SnTox3 infiltrated wheat at 48–102 hpi. All ten metabolite are unknown compounds present at higher levels in SnTox3-infiltrated wheat.

Metabolite extracts of four biological replicates of empty vector control- or SnTox3-infiltrated wheat at five time points post infiltration were analysed using Liquid Chromatography-Mass Spectrometry (LC-MS). PCA analysis of the processed data revealed that PC1 accounted for the largest variation in the data of 9.34% (Figure 7A). PC2 accounted for 5.6% of the variation within this dataset and best describes the differences between the control and SnTox3-infiltrated samples. The 10 mass features with the highest contribution to the variation seen in PC2 are detailed in Figure 7B.

Statistical analysis identified 31 mass features detected consistently across the time points and treatments to be significantly (*p* < 0.01) altered in their abundance in SnTox3-treated wheat relative to the control (Additional file 11). The MassHunter formula generator algorithm was used to generate empirical formulae for the mass features based on the abundances of carbon isotopes present in the molecular ion. Putative metabolite identifications were established by searching these empirical formulas against the publicly available MassBank, ReSpect and METLIN online metabolite databases. Putative metabolite identifications include the phenylpropanoids feruloylquinic acid and chlorogenic acid that increase at 48–102 hpi respectively. MS/MS data were generated for these mass features resulting in the identification of at least three fragments each matching those expected for these two compounds. Of particular interest was the metabolite that was putatively identified as the cyanogenic glycoside dhurrin. This compound is one of the few significant changes (*p* = 1.01 × 10^−11^) occurring as early as 12 hrs post SnTox3-infiltration.

## Discussion

Three effector proteins from the wheat pathogen *S. nodorum* have been identified recently, SnToxA, SnTox1 and SnTox3. With the exception of SnToxA (also present in *P. tritici-repentis*), these proteins are unique to *S. nodorum* and are of unknown function. In this study, we have taken the approach of analysing how the host responds to SnTox3 exposure at the transcript, protein and metabolite levels. It was not the intention of this manuscript to undertake a complete system biology analysis of this system and deliberately link the data from each of these approaches. Rather, these analyses were chosen for their complementarity and capacity to reveal different aspects of the interaction of the effector protein and the susceptible host. For example, the microarray approach provides a rapid overview of gene expression in a particular sample at a given time. However, previous studies have demonstrated that gene expression and protein abundance don’t always correlate and thus it was also important to consider protein abundances [33–35]. Lastly, we looked at the metabolome of the infiltrated wheat leaves to gain an understanding of the host metabolic status subsequent to SnTox3 infiltration in addition to identifying potential key defence compounds.

Metabolomic analysis revealed significant increases in hexoses in response to SnTox3. This in itself is intriguing, as it could have been expected that the significant down-regulation observed in photosynthesis would have lead to lower hexose levels. However, this reduction could be compensated by induction of respiratory metabolism and the oxidative pentose phosphate pathway (PPP) [36–38]. Induction of the latter was apparent at the transcriptional and translational level. Metabolomic analysis revealed an increase of intracellular ribose and fructose-6-phosphate suggesting that SnTox3-treatment may result in an increased flux through the non-oxidative phase of the PPP. Additionally, probe sets coding for glycolytic enzymes showed induced expression levels. The SnTox3 induced accumulation of sucrose, fructose and glucose are likely to fuel these reactions. The TCA cycle intermediates malic acid and citric acid were more abundant in leaf and apoplast samples at 72 hpi.

It has been previously described that elevated hexose levels may trigger the induction of plant defence [39]. Increased hexose levels have also been observed in incompatible biotrophic interactions suggesting that sugars fuel successful plant defence responses likely through the production of secondary metabolites [40,41]. Increases in hexoses have been shown to up-regulate expression of genes involved in phenylpropanoid biosynthesis [42,43]. Indeed, our metabolite profiling demonstrated the accumulation of the phenylpropanoids feruloylquinic acid and chlorogenic acid. Chlorogenic acid has inhibitory effects against bacterial and fungal pathogens and is suggested to interfere with cell membranes [44,45]. The activity of feruloylquinic acid may lie in the hydrolysis of this ester to release quinic acid and ferulic acid. Ferulic acid is implicated in resistance to a number of fungal pathogens including *Fusarium graminearum* [46–48]*.* SnTox3 also induced an increase in quinic acid, although ferulic acid was not detected.

The putatively identified cyanogenic glycoside dhurrin also increased in response to SnTox3. This was one of the first secondary metabolite changes identified and therefore has significant potential to impact the outcome of an *S. nodorum* infection. Cyanogenic glycosides are found in over 2,500 plant species and act as successful herbivore deterrents and are also correlated with pathogen resistance [49–52]. Compartmentalized within host cells to avoid self-toxicity, cyanogenic glycosides are cleaved upon tissue disruption releasing toxic hydrogen cyanide (HCN). Interestingly, intact cyanogenic glycosides have also demonstrated activity in barley where they are implicated in host recognition by the pathogen *Blumeria graminis* f. sp. *hordei* [53]. Many crop plants are cyanogenic including sorghum, wheat, barley, and rice [54]. Dhurrin was originally discovered in sorghum [55] and has also been reported in other members of the Poacaeae including *Triticum aestivum* [56] but there have been no reports of its activity or presence in wheat since.

Signalling elements typically observed during plant defence were also evident subsequent to SnTox3 infiltration. Transcriptome analysis revealed that several probe sets coding for MAP kinases were induced by SnTox3. The role of MAP kinases in plant defence responses has been studied extensively and they play a crucial role in the establishment of PAMP-triggered immunity [1,57]. Among them is TaMPK3, whose expression is significantly induced at 24 hpi and 48 hpi (fold change 8.0 and 6.3, respectively). TaMPK3 has previously been shown to be transcriptionally induced by the necrotrophic effector PtrToxA [15] and to be activated during interactions with the necrotrophic fungal pathogen *Zymoseptoria tritici* [58]. Interestingly, TaMPK3 expression precedes necrotic symptom development and shows peak expression levels just before the appearance of macroscopic symptoms for SnTox3 and PtrToxA infiltrations as well as the *Z. tritici* infection suggesting that it is involved in the regulation of PCD onset.

A striking observation from both the transcriptomics and proteomics data was the up-regulation of methionine metabolism subsequent to SnTox3 infiltration. One consequence of this is likely to be an increase in the levels of homocysteine and SAM. SAM is a direct precursor for ethylene biosynthesis and primary methyl donor for a variety of defence-related metabolites. Its methyl group is consumed in the biosynthesis of phenylpropanoids such as feruloylquinic acid and chlorogenic acid [59]. Phenylpropanoids can also act as phytoalexins, phytoanticipans and signalling molecules inducing plant defence gene expression [60,61]. Additionally, decarboxylated SAM donates propylamine groups during biosynthesis of polyamines including spermine and spermidine [62]. Transgenic lines of tomato overproducing spermidine are more susceptible to the necrotrophic fungus *B. cinerea* [63] whilst spermine has been shown to induces expression of PR genes [64].

It has also been reported that homocysteine levels are higher in potato infected with *Phytopthora infestans*, especially in susceptible potato genotypes at the later stages of the disease. Furthermore, infiltration of leaves with homocysteine results in quicker and more severe disease progression in susceptible plants suggesting that homocysteine is playing a role in accelerating host cell death [65] and highlighting the potential role for homocysteine and/or potential downstream responses for influencing plant-pathogen interactions.

Together, the above examples indicate that an increase in the metabolism of methionine through homocysteine plays a role in host defence, possibly through multiple mechanisms. Our data here also suggests such a role for the *S. nodorum* –wheat interaction. The elevation of many of the transcripts and proteins associated with the methionine cycle after SnTox3 treatment correlates with the increase in the other observed defence responses (e.g. PR proteins and secondary metabolism). Furthermore, the ability *S. nodorum* to cause disease was hindered in the presence of increased levels of homocysteine suggesting that it, or its metabolism, plays an as yet undiscovered role role in host defence.

These data though raise the question of how does *S. nodorum* deal with these defence responses during infection? There are two possibilities. Firstly, the rate of the infection outpaces the defence responses allowing the pathogen to sporulate prior to inhibition. It could perhaps be speculated that the increasing defence responses are a signal for the pathogen to sporulate. Another possibility may be that the pathogen suppresses these defence responses during infection, as commonly occurs in biotrophic and hemi-biotrophic pathogens. A previous study by our laboratory has evidence to support this. During exposure to purified SnToxA, high levels of serotonin were seen to accumulate in susceptible wheat lines [17]. It was demonstrated that serotonin strongly inhibited the ability of the pathogen to asexually sporulate. However, when the same lines were infected with *S. nodorum*, the serotonin levels were 10-fold decreased suggesting that the pathogen has mechanisms for suppressing plant defences during infection. The mechanisms behind this are currently unclear, however it wouldn’t be unexpected that one (or more) of the many small secreted proteins encoded in the *S. nodorum* genome could have a role in defence suppression. Indeed recent studies have identified that *Botrytis cinerea* suppresses host immunity by hijacking host RNA interference pathways using small RNAs [66].

Comparable host analyses studies have been previously published for ToxA from both *S. nodorum* and *P. tritici-repentis* [15–17,67]. A comparison of the SnToxA and SnTox3 datasets revealed several significant differences including signalling and primary metabolism changes in the host. There is also evidence to suggest that whilst both effectors may lead to collapse of photosynthesis, they do so through different means. SnTox3 leads to down-regulation of the light reaction (particularly photosystem I, ATP synthase) whilst SnToxA has a higher impact on the Calvin cycle potentially leading to feedback inhibition of photosynthesis [68].

Perhaps one of the most interesting differences when comparing the plant response to SnTox3 and SnToxA was on secondary metabolism. SnToxA strongly induced the tryptophan pathway as evident from microarray analysis [15]. Metabolome analysis detected significant increases in tryptophan, serotonin, two serotonin derivatives and the benzoxazinoid 6-methoxy-2-benzoxazolinone (MBOA) synthesized from the tryptophan precursor indole-3-glycerolphosphate [17]. In contrast to this, SnTox3 infiltration resulted in no increase in any of the tryptophan pathway metabolites and this was consistent with microarray data showing a significantly lower number of tryptophan pathway genes induced compared to SnToxA. Instead SnTox3 appears to induce the phenylpropanoid pathway as evident from our microarray analysis and the detection of the secondary metabolites feruloylquinic acid and chlorogenic acid suggesting that whilst SnToxA and SnTox3 both result in host cell death in susceptible wheat lines, each interaction is causal to other independent events, possibly indicative of other functions.

In conclusion, these data have identified many interesting pathways and processes in the host associated with exposure to the SnTox3 protein and highlight the almost paradoxical nature of the wheat-*S. nodorum* this interaction. The necrotrophic effectors secreted by *S. nodorum* result in host cell death through their interaction with dominant susceptibility loci. The cell death provides the environment for the necrotroph to derive nutrients and thrive prior to asexual development. However the effectors studied thus far also trigger a strong defence response (probably due to the host cell death or events leading to it). Studies are now underway to better understand how the pathogen copes with and/or suppresses the accumulation of defence proteins and metabolites. However we now need to functionally characterise some of these processes and determine precisely what role they have in disease.

## Conclusions

The study of necrotrophic pathogens has come-of-age. Until recently, necrotrophs were often considered crude and simplistic pathogens that were reliant on a battery of lytic enzymes to degrade host tissue for nutrient release. However, we now understand that some necrotrophic pathogens are more complex and subtle than originally thought. Seminal studies on victorin from *Cochliobolus victoriae* and also rapid progress on ToxA from *S. nodorum* and *P. tritici-repentis* have highlighted the gene-for-gene nature that exists in these interactions [6,7,20]. We now understand that these effectors have evolved to be recognised in order to facilitate pathogen growth *in planta*.

This study advances this necrotrophic gene-for-gene concept by revealing how wheat responds to exposure to the SnTox3 effector from *S. nodorum*. As the pathogen is strictly host specific for wheat, we are limited in this system in terms of resources available in dissecting the host in this interaction (e.g. T-DNA lines etc. aren’t available). Consequently studies such as this are important in building our fundamental base of understanding diseases such as this on non-model hosts. The success of this approach has been demonstrated through the recent discovery of the phyotalexin serotonin [17].

It is thought that the primary role of these effectors (SnTox3 and ToxA) is to induce cell death through the stimulation of existing defence pathways. The recent discovery of the Tsn1, an R-like susceptibility gene for ToxA supports this theory [14]. However, whilst the pathogen thrives from the nutrient release resulting from host cell death, it must also survive these resulting defence responses. Indeed, a previous study from this laboratory has identified that *S. nodorum* is able to limit the accumulation of serotonin induced upon ToxA exposure [17]. Studies are now underway to dissect the pathogen mechanisms for coping with these defence responses during infection.

## Additional files

Additional file 1:
**Placement of samples in runs.** T3 denotes TOX3-infiltrated samples, EV stands for Empty Vector _ infiltrated samples, with the subsequent numbers showing the time and sample, e.g. T348B is a TOX3-infiltrated sample B after 48 hours. Additional file 2:
**iTRAQ ratio compression and biological FDR estimation.**
Additional file 3:
**Overlap of differentially regulated probes ets in Tox3- and ToxA- infiltrated plants.**
Additional file 4:
**Summary of all probe sets differentially regulated upon SnTox3 infiltration.**
Additional file 5:
**Differentially expressed kinases and transcription factors.**
Additional file 6:
**Summary of all differentiall regulated proteins in response to SnTo3 treatment.**
Additional file 7:
***S. nodorum*** **in vitro growth assay in the presence of various concentrations of homocysteine in Minimal media.** Radial growth was measured at 18 days post inoculation. n = 3. Additional file 8:
**GC-MS analysis of SnTox3 infiltrated wheat (cv. BG220 Snn3 locus) at 4–72 hrs post infiltration.**
Additional file 9:
**GC-MS analysis of apoplast extracted from SnTox3 infiltrated wheat (cv. BG220 Snn3 locus) at 4–72 hrs post infiltration.**
Additional file 10:
**Metabolite analysis of apoplast extracted from SnTox3 infiltrated wheat. **
Additional file 11:
**LC-MS analysis of SnTox3 infiltrated wheat (cv. BG220 Snn3 locus) at 4–102 hrs post infiltration.**

## References

[1] Jones JDG, Dangl JL (2006). The plant immune system. Nature.

[2] Chisholm ST, Coaker G, Day B, Staskawicz BJ (2006). Host-microbe interactions: shaping the evolution of the plant immune response. Cell.

[3] Liu Z, Faris JD, Oliver RP, Tan KC, Solomon PS, McDonald MC, McDonald BA, Nunez A, Lu S, Rasmussen JB, Friesen TL (2009). SnTox3 acts in effector triggered susceptibility to induce disease on wheat carrying the Snn3 gene. PLoS Pathog.

[4] Wolpert TJ, Macko V, Acklin W, Arigoni D: **Molecular features affecting the biological activity of the host-selective toxins from*****Cochliobolus victoriae*****.** *Plant Physiol* 1988, **88:**37–41. 10.1104/pp.88.1.37 PMC1055521 16666275

[5] Lorang JM, Sweat TA, Wolpert TJ (2007). Plant disease susceptibility conferred by a “resistance” gene. Proc Natl Acad Sci U S A.

[6] Lorang J, Kidarsa T, Bradford CS, Gilbert B, Curtis M, Tzeng SC, Maier CS, Wolpert TJ (2012). Tricking the guard: exploiting plant defense for disease susceptibility. Science.

[7] Ciuffetti LM, Manning VA, Pandelova I, Betts MF, Martinez JP: **Host-selective toxins, Ptr ToxA and Ptr ToxB, as necrotrophic effectors in the*****Pyrenophora tritici-repentis*****-wheat interaction.** *New Phytol* 2010, **187:**911–919. 10.1111/j.1469-8137.2010.03362.x 20646221

[8] Ballance GM, Lamari L, Bernier CC: **Purification and characterization of a host-selective necrosis toxin from*****Pyrenophora tritici-repentis*****.** *Physiol Mol Plant Pathol* 1989, **35:**203–213.

[9] Ciuffetti LM, Tuori RP, Gaventa JM (1997). A single gene encodes a selective toxin causal to the development of tan spot of wheat. Plant Cell.

[10] Tuori RP, Wolpert TJ, Ciuffetti LM (2000). Heterologous expression of functional Ptr ToxA. Mol Plant-Microbe Interact.

[11] Manning VA, Ciuffetti LM: **Localization of Ptr ToxA produced by*****Pyrenophora tritici-repentis*****reveals protein import into wheat mesophyll cells.** *Plant Cell* 2005, **17:**3203–3212. 10.1105/tpc.105.035063 PMC1276038 16199615

[12] Manning VA, Chu AL, Steeves JE, Wolpert TJ, Ciuffetti LM: **A host-selective toxin of*****Pyrenophora tritici-repentis*****, Ptr ToxA, induces photosystem changes and reactive oxygen species accumulation in sensitive wheat.** *Mol Plant-Microbe Interact* 2009, **22:**665–676. 10.1094/MPMI-22-6-0665 19445591

[13] Manning VA, Hardison LK, Ciuffetti LM (2007). Ptr ToxA interacts with a chloroplast-localized protein. Mol Plant-Microbe Interact.

[14] Faris JD, Zhang Z, Lu H, Lu S, Reddy L, Cloutier S, Fellers JP, Meinhardt SW, Rasmussen JB, Xu SS, Oliver RP, Simons KJ, Friesen TL (2010). A unique wheat disease resistance-like gene governs effector-triggered susceptibility to necrotrophic pathogens. Proc Natl Acad Sci U S A.

[15] Pandelova I, Betts MF, Manning VA, Wilhelm LJ, Mockler TC, Ciuffetti LM (2009). Analysis of transcriptome changes induced by Ptr ToxA in wheat provides insights into the mechanisms of plant susceptibility. Mol Plant.

[16] Vincent D, Du Fall LA, Livk A, Mathesius U, Lipscombe RJ, Oliver RP, Friesen TL, Solomon PS: **A functional genomics approach to dissect the mode of action of the*****Stagonospora nodorum*****effector protein SnToxA in wheat.** *Mol Plant Pathol* 2012, **13:**467–482. 10.1111/j.1364-3703.2011.00763.x PMC6638714 22111512

[17] Du Fall LA, Solomon PS (2013). The necrotrophic effector SnToxA induces the synthesis of a novel phytoalexin in wheat. New Phytol.

[18] Strelkov SE, Lamari L: **Host-parasite interactions in tan spot*****Pyrenophara tritici-repentis*****of wheat.** *Can J Plant Pathol* 2003, **25:**339–349.

[19] Friesen TL, Faris JD, Solomon PS, Oliver RP (2008). Host-specific toxins: effectors of necrotrophic pathogenicity. Cell Microbiol.

[20] Oliver RP, Solomon PS (2010). New developments in pathogenicity and virulence of necrotrophs. Curr Opin Plant Biol.

[21] Oliver RP, Friesen TL, Faris JD, Solomon PS: ***Stagonospora nodorum*****: from pathology to genomics and host resistance.** *Annu Rev Phytopathol* 2012, **50:**23–43. 10.1146/annurev-phyto-081211-173019 22559071

[22] Friesen TL, Stukenbrock EH, Liu Z, Meinhardt S, Ling H, Faris JD, Rasmussen JB, Solomon PS, McDonald BA, Oliver RP (2006). Emergence of a new disease as a result of interspecific virulence gene transfer. Nat Genet.

[23] Liu Z, Zhang Z, Faris JD, Oliver RP, Syme R, McDonald MC, McDonald BA, Solomon PS, Lu S, Shelver WL, Xu S, Friesen TL: **The cysteine rich necrotrophic effector SnTox1 produced by*****Stagonospora nodorum*****triggers susceptibility of wheat lines harboring Snn1.** *PLoS Pathog* 2012, **8:**e1002467. 10.1371/journal.ppat.1002467 PMC3252377 22241993

[24] IpCho SVS, Hane JK, Antoni EA, Ahren D, Henrissat B, Friesen TL, Solomon PS, Oliver RP: **Transcriptome analysis of*****Stagonospora nodorum*****: gene models, effectors, metabolism and pantothenate dispensability.** *Mol Plant Pathol* 2011, **13:**531–545. 10.1111/j.1364-3703.2011.00770.x PMC6638697 22145589

[25] Zhang Z, Friesen TL, Xu SS, Shi G, Liu Z, Rasmussen JB, Faris JD: **Two putatively homoeologous wheat genes mediate recognition of SnTox3 to confer effector-triggered susceptibility to*****Stagonospora nodorum*****.** *Plant J* 2011, **65:**27–38. 10.1111/j.1365-313X.2010.04407.x 21175887

[26] Solomon PS, Rybak K, Trengove RD, Oliver RP: **Investigating the role of calcium/calmodulin-dependent protein kinases in*****Stagonospora nodorum*****.** *Mol Microbiol* 2006, **62:**367–381. 10.1111/j.1365-2958.2006.05380.x 17020577

[27] Eisenhart C (1947). The assumptions underlying the analysis of variance. Biometrics.

[28] Tamhane AC, Dunlop DD (2000). Statistics and Data Analysis: From Elementary to Intermediate.

[29] Usadel B, Poree F, Nagel A, Lohse M, Czedik-Eysenberg A, Stitt M (2009). A guide to using MapMan to visualize and compare omics data in plants: a case study in the crop species, maize. Plant Cell Environ.

[30] Pascovici D, Gardiner DM, Song X, Breen E, Solomon PS, Keighley T, Molloy MP (2013). Coverage and consistency: bioinformatics aspects of the analysis of multi-run iTRAQ experiments of wheat leaves. J Proteome Res.

[31] Solomon PS, Oliver RP: **The nitrogen content of the tomato leaf apoplast increases during infection by*****Cladosporium fulvum*****.** *Planta* 2001, **213:**241–249. 10.1007/s004250000500 11469589

[32] Mahoney DW, Therneau TM, Heppelmann CJ, Higgins L, Benson LM, Zenka RM, Jagtap P, Nelsestuen GL, Bergen IHR, Oberg AL (2011). Relative quantification: characterization of bias, variability and fold changes in mass spectrometry data from iTRAQ-labeled peptides. J Proteome Res.

[33] Gygi SP, Rochon Y, Franza BR, Aebersold R (1999). Correlation between protein and mRNA abundance in yeast. Mol Cell Biol.

[34] Foss EJ, Radulovic D, Shaffer SA, Ruderfer DM, Bedalov A, Goodlett DR, Kruglyak L (2007). Genetic basis of proteome variation in yeast. Nat Genet.

[35] Ghazalpour A, Bennett B, Petyuk VA, Orozco L, Hagopian R, Mungrue IN, Farber CR, Sinsheimer J, Kang HM, Furlotte N, Park CC, Wen PZ, Brewer H, Weitz K, Camp Li DG, Pan C, Yordanova R, Neuhaus I, Tilford C, Siemers N, Gargalovic P, Eskin E, Kirchgessner T, Smith DJ, Smith RD, Lusis AJ (2011). Comparative analysis of proteome and transcriptome variation in mouse. PLoS Genet.

[36] Bilgin DD, Zavala JA, Zhu J, Clough SJ, Ort DR, DeLucia EH (2010). Biotic stress globally downregulates photosynthesis genes. Plant Cell Environ.

[37] Bolton MD (2009). Primary metabolism and plant defense-fuel for the fire. Mol Plant-Microbe Interact.

[38] Kangasjärvi S, Neukermanns J, Li S, Aro E-M, Noctor G (2012). Photosynthesis, photorespiration, and light signalling in defence responses. J Exp Bot.

[39] Moghaddam- Boulori MRB, Van Den Ende W (2012). Sugars and plant innate immunity. J Exp Bot.

[40] Swarbrick PJ, Schulze-Lefert P, Scholes JD (2006). Metabolic consequences of susceptibility and resistance (race-specific and broad-spectrum) in barley leaves challenged with powdery mildew. Plant Cell Environ.

[41] Herbers K, Meuwly P, Frommer WB, Metraux JP, Sonnewald U (1996). Systemic acquired resistance mediated by the ectopic expression of invertase: possible hexose sensing in the secretory pathway. Plant Cell.

[42] Lloyd JC, Zakhleniuk OV (2004). Responses of primary and secondary metabolism to sugar accumulation revealed by microarray expression analysis of the Arabidopsis mutant, pho3. J Exp Bot.

[43] Solfanelli C, Poggi A, Loreti E, Alpi A, Perata P (2006). Sucrose-specific induction of the anthocyanin biosynthetic pathway in Arabidopsis. Plant Physiol.

[44] Sung WS, Lee DG (2010). Antifungal action of chlorogenic acid against pathogenic fungi, mediated by membrane disruption. Pure Appl Chem.

[45] López-Gresa MP, Torres C, Campos L, Lisón P, Rodrigo I, Bellés JM, Conejero V: **Identification of defence metabolites in tomato plants infected by the bacterial pathogen*****Pseudomonas syringae*****.** *Environ Exp Bot* 2011, **74:**216–218.

[46] Cabrera HM, Munoz O, Zuniga GE, Corcuera LJ, Argandona VH (1995). Changes in ferulic acid and lipid content in aphid-infested barley. Phytochemistry.

[47] Bily AC, Reid LM, Taylor JH, Johnston D, Malouin C, Burt AJ, Bakan B, Regnault-Roger C, Pauls KP, Arnason JT, Philogene BJR: **Dehydrodimers of ferulic acid in maize grain pericarp and aleurone: resistance factors to*****Fusarium graminearum*****.** *Phytopathology* 2003, **93:**712–719. 10.1094/PHYTO.2003.93.6.712 18943058

[48] Santiago R, Butron A, Arnason JT, Reid LM, Souto XC, Malvar RA: **Putative role of pith cell wall phenylpropanoids in*****Sesamia nonagrioides*****(Lepidoptera: Noctuidae) resistance.** *J Agric Food Chem* 2006, **54:**2274–2279. 10.1021/jf0524271 16536607

[49] Tattersall DB, Bak S, Jones PR, Olsen CE, Nielsen JK, Hansen ML, Høj PB, Møller BL (2001). Resistance to an herbivore through engineered cyanogenic glucoside synthesis. Science.

[50] Bak S, Paquette SM, Morant M, Morant AV, Saito S, Bjarnholt N, Zagrobelny M, Jørgensen K, Osmani S, Simonsen HT, Perez RS, Van Heeswijick TB, Jørgensen B, Møller BL (2006). Cyanogenic glycosides: a case study for evolution and application of cytochromes P450. Phytochem Rev.

[51] Iriti M, Faoro F (2009). Chemical diversity and defence metabolism: How plants cope with pathogens and ozone pollution. Int J Mol Sci.

[52] Du Fall LA, Solomon PS (2011). Role of cereal secondary metabolites involved in mediating the outcome of plant-pathogen interactions. Metabolites.

[53] Nielsen KA, Olsen CE, Pontoppidan K, Moller BL (2002). Leucine-derived cyano glucosides in barley. Plant Physiol.

[54] Jones DA (1998). Why are so many food plants cyanogenic?. Phytochemistry.

[55] Møller BL, Conn EE: **The biosynthesis of cyanogenic glucosides in higher plants. N-Hydroxytyrosine as an intermediate in the biosynthesis of dhurrin by*****Sorghum bicolor*****(Linn) Moench.** *J Biol Chem* 1979, **254:**8575–8583. 468842

[56] Gorz HJ, Haskins FA, Dam R, Vogel KP: **Dhurrin in*****Sorghastrum nutans*****.** *Phytochemistry* 1979, **18:**2024.

[57] Pitzschke A, Schikora A, Hirt H (2009). MAPK cascade signalling networks in plant defence. Curr Opin Plant Biol.

[58] Rudd JJ, Keon J, Hammond-Kosack KE: **The wheat mitogen-activated protein kinases TaMPK3 and TaMPK6 are differentially regulated at multiple levels during compatible disease interactions with*****Mycosphaerella graminicola*****.** *Plant Physiol* 2008, **147:**802–815. 10.1104/pp.108.119511 PMC2409019 18441220

[59] Moffatt BA, Weretilnyk EA (2001). Sustaining S-adenosyl-L-methionine-dependent methyltransferase activity in plant cells. Physiol Plant.

[60] Leiss KA, Maltese F, Choi YH, Verpoorte R, Klinkhamer PGL (2009). Identification of chlorogenic acid as a resistance factor for thrips in chrysanthemum. Plant Physiol.

[61] Dixon RA, Achnine L, Kota P, Liu CJ, Reddy MSS, Wang L (2002). The phenylpropanoid pathway and plant defence - A genomics perspective. Mol Plant Pathol.

[62] Sauter M, Moffatt B, Saechao MC, Hell R, Wirtz M (2013). Methionine salvage and S-adenosylmethionine: essential links between sulfur, ethylene and polyamine biosynthesis. Biochem J.

[63] Nambeesan S, AbuQamar S, Laluk K, Mattoo AK, Mickelbart MV, Ferruzzi MG, Mengiste T, Handa AK: **Polyamines attenuate ethylene-mediated defense responses to abrogate resistance to*****Botrytis cinerea*****in tomato.** *Plant Physiol* 2012, **158:**1034–1045. 10.1104/pp.111.188698 PMC3271740 22128140

[64] Yamakawa H, Kamada H, Satoh M, Ohashi Y (1998). Spermine is a salicylate-independent endogenous inducer for both tobacco acidic pathogenesis-related proteins and resistance against tobacco mosaic virus infection. Plant Physiol.

[65] Arasimowicz-Jelonek M, Floryszak-Wieczorek J, Gzyl J, Chmielowska-Bak J (2013). Homocysteine over-accumulation as the effect of potato leaves exposure to biotic stress. Plant Physiol Biochem.

[66] Weiberg A, Wang M, Lin F-M, Zhao H, Zhang Z, Kaloshian I, Huang H-D, Jin H (2013). Fungal small RNAs suppress plant immunity by hijacking host RNA interference pathways. Science.

[67] Adhikari TB, Bai J, Meinhardt SW, Gurung S, Myrfield M, Patel J, Ali S, Gudmestad NC, Rasmussen JB: **Tsn1-mediated host responses to ToxA from*****Pyrenophora tritici-repentis*****.** *Mol Plant-Microbe Interact* 2009, **22:**1056–1068. 10.1094/MPMI-22-9-1056 19656041

[68] Paul MJ, Pellny TK (2003). Carbon metabolite feedback regulation of leaf photosynthesis and development. J Exp Bot.

