# Supplementary material for: The necrotrophic effector protein SnTox3 re-programs metabolism and elicits a strong defence response in susceptible wheat leaves
Source: BMC Plant Biol. 2014 Aug 15;14:215. doi: 10.1186/s12870-014-0215-5 (PMC4243954; doi:10.1186/s12870-014-0215-5)
Supplement: Additional file 2: — iTRAQ ratio compression and biological FDR estimation. [file 12870_2014_215_MOESM2_ESM.pdf]

Additional file 2

iTRAQ ratio compression and biological FDR estimation

iTRAQ ratio compression is widely reported in several highly regarded references such as [1],[2], [3] and many others. Mahoney et al [3] emphasized that, because lower abundance peptides usually exhibit more variablilty, fold changes *alone* cannot be employed, hence the necessity for additional statistical significance measures such as p-values or confidence intervals. While this reference is often misquoted to say that “fold changes cannot be applied”, what it actually shows is that *additionally* to fold changes *measures of statistical significance* (p-values, CI’s) are needed because of the increased variability at low abundance. Mahoney et al show that in their hands a true fold change of 2 corresponds to approximately a detected fold change of 1.5, and a true fold change of 1.5 to approximately 1.3 ([3] Figure 2) – we add that ratio compression is known to depend on sample complexity, hence such figures are an indication only.

The influential reference of Karp et al [2] found that, in their experimental and analytic setup, a ”±1.1 fold-change threshold encompassed 95% of the experimental variation”, and consequently “low changes in expression will be detectable in a pair-wise comparison, although the researcher will need to assess such a change is biologically significant.”([2] Sec. 3.3.3). In their setup thus, fold changes of ±1.2 are greater than the experimental variation and could be detected, though the attached warnings of course apply.

A cutoff in the range of 1.2- 1.25 is frequently employed in the literature at large (for example [4],[5]), commonly employed by our group ([6], [7]), and in the context of this dataset corresponds to approximately a normalized z-score of 1 for the fold-change data (1 standard deviation away from the mean of log fold changes). In this current work, we use three biological replicates in each condition on separate iTRAQ runs, and we use t-test p-values as measures of significance. We believe this is a strict scenario particularly given the confirmatory purpose of this data alongside the transcriptomics results.

Additionally, in the subsequent section we evaluate the impact of such cut-offs on differential expression of biological replicates to demonstrate that the resulting false discovery rate is low.

Estimation of “Biological FDR”

With our given experimental design described in the supplementary notes, the following biological replicates are placed on the same run: Tox3 12 A and B, EV 12 B and C, Tox3 24 A and B, EV 24 B and C, Tox3 48 A and B, EV 48 B and C, Tox3 72 A and B and EV 72 B and C. In each case, we identify differential expression by our commonly employed criterion of ProteinPilot ratio > 1.2 or < 0.83, ProteinPilot p-value for the respective protein < 0.05 (no multiple testing corrections). The ProteinPilot p-values are based on the peptide distribution and included in the “ProteinSummary” files for each iTRAQ run.

Then Biological FDR = (N=number of proteins found differentially expressed between the two replicates)/(Total number of proteins quantitated in the respective run)

We plot the protein ratios and p-values in a volcano plot for each pair of biological replicates (Supplementary Figure), and attach the resulting rates in the table below. The “Biological FDR” is around 1% for most pairs of replicates. For technical replicates the FDR would be even lower, but since these biological replicates are pools of plants in different pots in the same conditions, we would likewise expect the variabiliy to be quite low.

These tables show that the employed criteria are quite conservative despite the seemingly low fold change cut-off; they also support the statement that “fold changes ***alone*** cannot be employed”, but supplemented by measure of consistency they can provide low false discovery rates.

Table Supplementary: Placement of samples in runs.

	114	115	116	117
**Run 1**	Pool	T312A	T312B	EV12A
**Run 2**	EV12B	pool	EV12C	T312C
**Run 3**	T324A	T324B	pool	EV24A
**Run 4**	EV24B	EV24C	T324C	pool
**Run 5**	Pool	T348A	T348B	EV48A
**Run 6**	EV48B	pool	EV48C	T348C
**Run 7**	T372A	T372B	pool	EV72A
**Run 8**	EV72B	EV72C	T372C	pool

Run	Quantitated	DE between biological replicates	Biological FDR
1	1429	14	0.98%
2	1079	17	1.58%
3	1124	12	1.07%
4	900	5	0.56%
5	767	25	3.26%
6	1179	8	0.68%
7	898	17	1.89%
8	986	0	0.00%

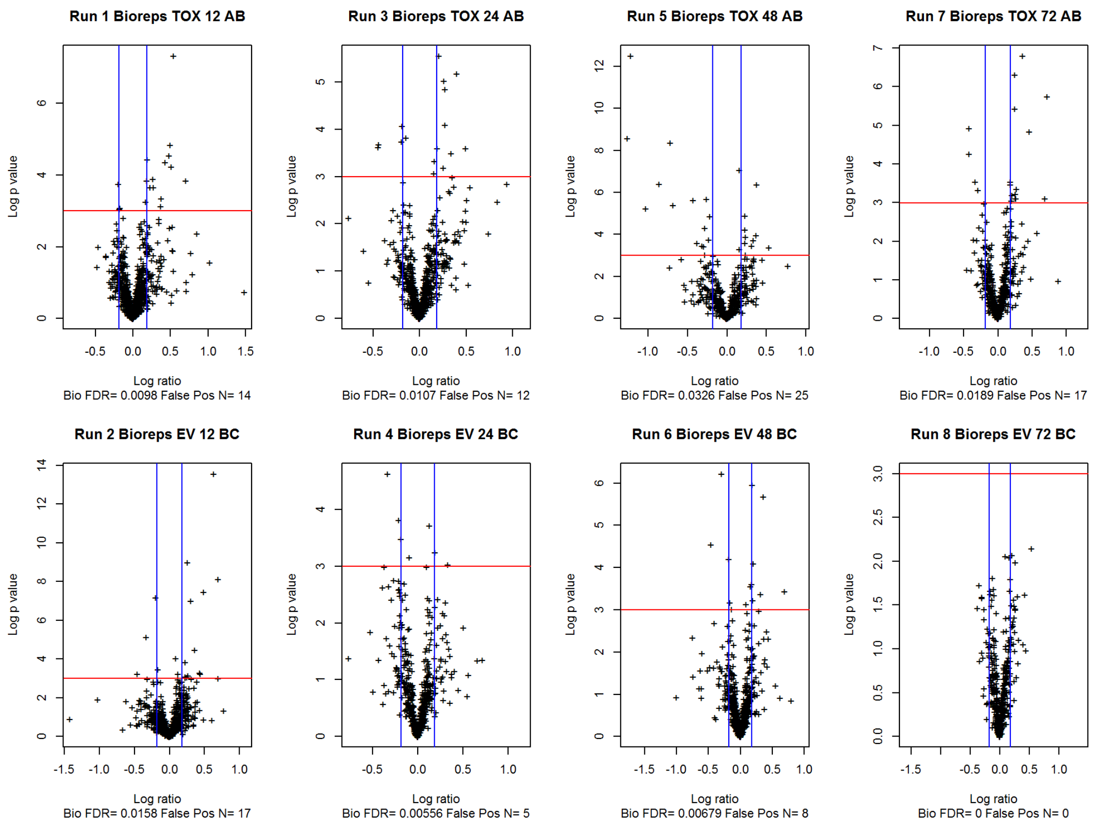

References

1. Ow SY, Salim M, Noirel J, Evans C, Rehman I, Wright PC: **iTRAQ Underestimation in Simple and Complex Mixtures: "The Good, the Bad and the Ugly"**. *Journal of Proteome Research* 2009, **8**(11):5347-5355.
2. Karp NA, Huber W, Sadowski PG, Charles PD, Hester SV, Lilley KS: **Addressing accuracy and precision issues in iTRAQ quantitation**. *Molecular & Cellular Proteomics* 2010, **9**(9):1885-1897.
3. Mahoney DW, Therneau TM, Heppelmann CJ, Higgins L, Benson LM, Zenka RM, Jagtap P, Nelsestuen GL, Bergen III HR, Oberg AL: **Relative quantification: characterization of bias, variability and fold changes in mass spectrometry data from iTRAQ-labeled peptides**. *Journal of Proteome Research* 2011, **10**(9):4325-4333.
4. Salim K, Kehoe L, Minkoff MS, Bilsland JG, Munoz-Sanjuan I, Guest PC: **Identification of differentiating neural progenitor cell markers using shotgun isobaric tagging mass spectrometry**. *Stem cells and development* 2006, **15**(3):461-470.

5. Seshi B: **An integrated approach to mapping the proteome of the human bone marrow stromal cell**. *PROTEOMICS* 2006, **6**(19):5169-5182.
6. Rhein V, Song X, Wiesner A, Ittner LM, Baysang G, Meier F, Ozmen L, Bluethmann H, Dröse S, Brandt U *et al*: **Amyloid-β and tau synergistically impair the oxidative phosphorylation system in triple transgenic Alzheimer's disease mice**. *Proceedings of the National Academy of Sciences* 2009, **106**(47):20057-20062.
7. Liang V, Ullrich M, Lam H, Chew Y, Banister S, Song X, Zaw T, Kassiou M, Götz J, Nicholas H: **Altered proteostasis in aging and heat shock response in C. elegans revealed by analysis of the global and de novo synthesized proteome**. *Cell Mol Life Sci* 2014:1-23.
