# Supplementary material for: The necrotrophic effector protein SnTox3 re-programs metabolism and elicits a strong defence response in susceptible wheat leaves
Source: BMC Plant Biol. 2014 Aug 15;14:215. doi: 10.1186/s12870-014-0215-5 (PMC4243954; doi:10.1186/s12870-014-0215-5)
Supplement: Additional file 3: — Overlap of differentially regulated probes ets in Tox3- and ToxA- infiltrated plants. [file 12870_2014_215_MOESM3_ESM.pdf]

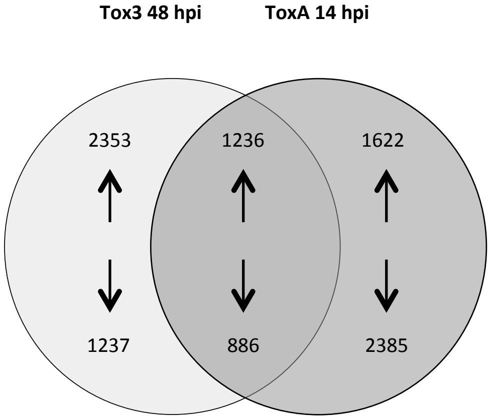

Additional file 3. Overlap of differentially regulated probe sets in Tox3- and ToxA-infiltrated plants
