# Supplementary material for: The necrotrophic effector protein SnTox3 re-programs metabolism and elicits a strong defence response in susceptible wheat leaves
Source: BMC Plant Biol. 2014 Aug 15;14:215. doi: 10.1186/s12870-014-0215-5 (PMC4243954; doi:10.1186/s12870-014-0215-5)
Supplement: Additional file 7: — S. nodorum in vitro growth assay in the presence of various concentrations of homocysteine in Minimal media. Radial growth was measured at 18 days post inoculation. n = 3. [file 12870_2014_215_MOESM7_ESM.pdf]

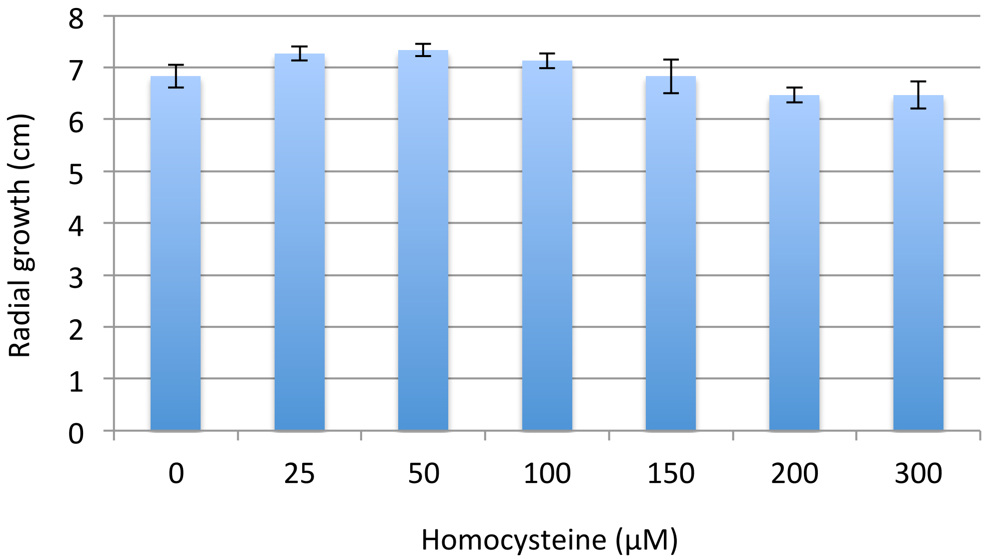

Additional file 7. *S. nodorum in vitro* growth assay in the presence of various concentrations of homocysteine in Minimal media. Radial growth was measured at 18 days post inoculation. n=3.
