# Supplementary figures and images for: The necrotrophic effector protein SnTox3 re-programs metabolism and elicits a strong defence response in susceptible wheat leaves
Source: BMC Plant Biol. 2014 Aug 15;14:215. doi: 10.1186/s12870-014-0215-5 (PMC4243954; doi:10.1186/s12870-014-0215-5)

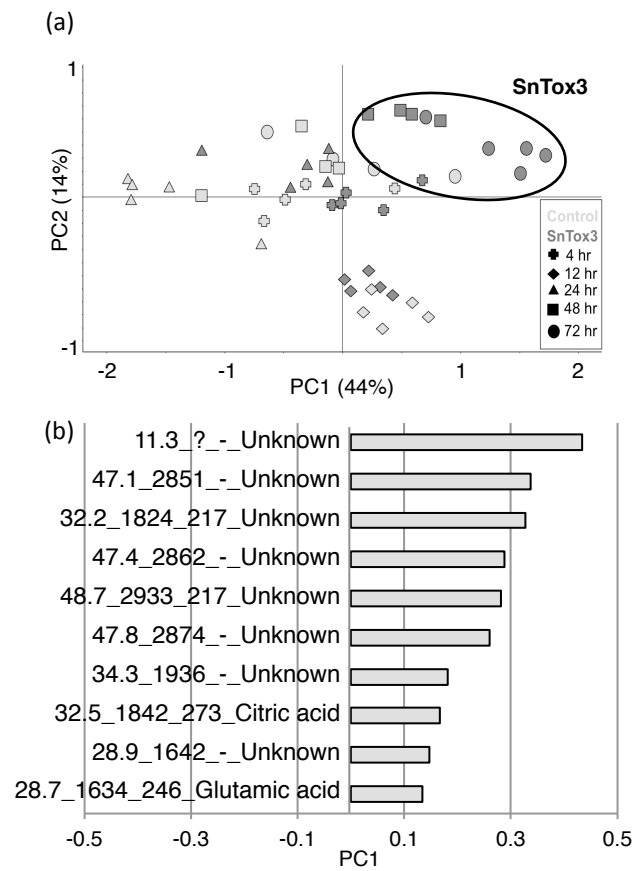

**Additional file 10.** Metabolite analysis of apoplast extracted from SnTox3 infiltrated wheat.

Supplement: Additional file 10: — Metabolite analysis of apoplast extracted from SnTox3 infiltrated wheat. [file 12870_2014_215_MOESM10_ESM.pdf]
